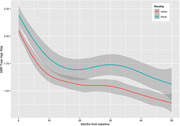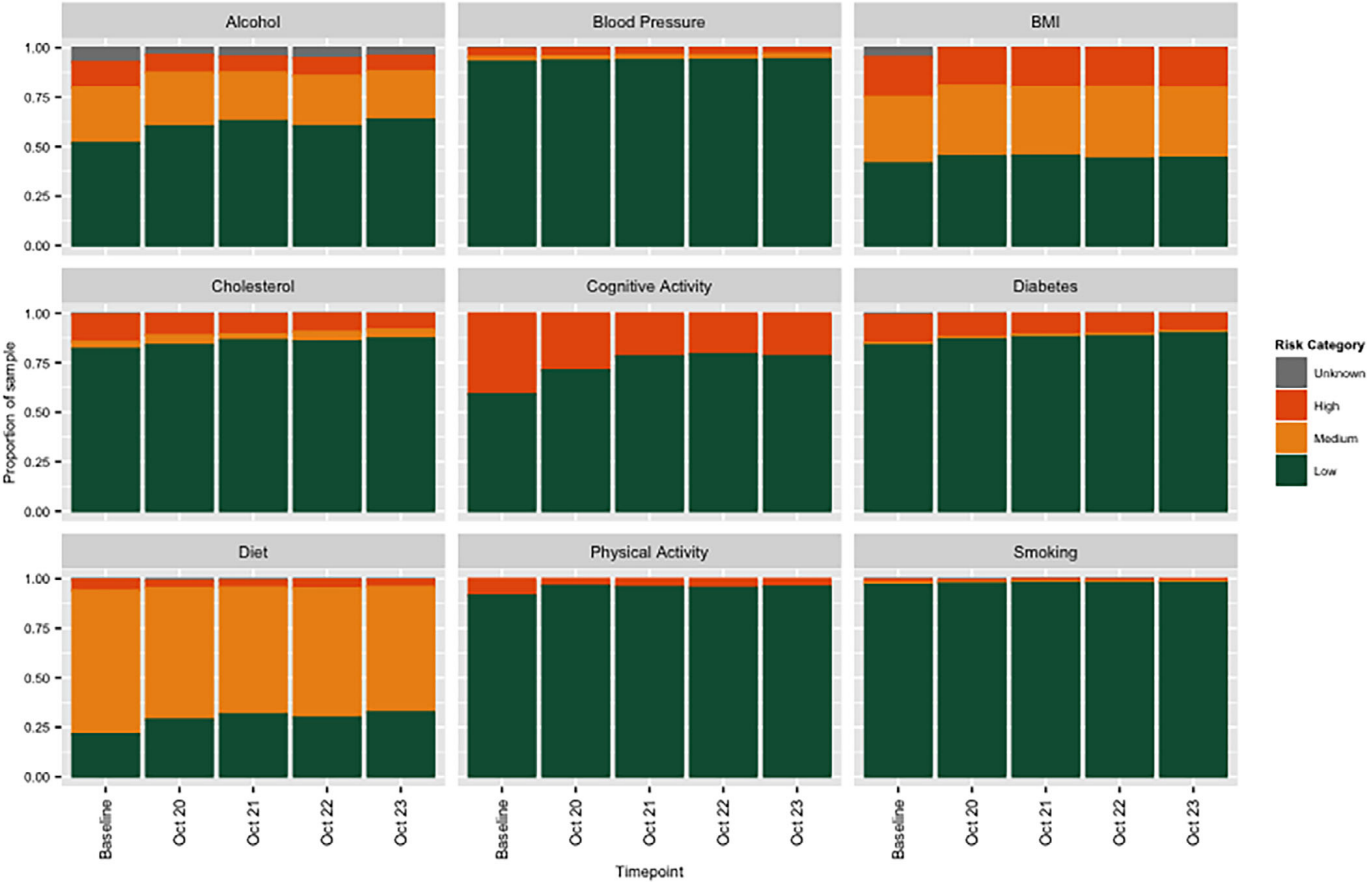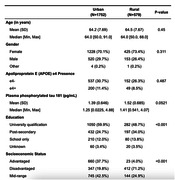# Rural‐urban improvements in modifiable risk factors across Tasmania from an online, public health dementia risk reduction initiative: data from ISLAND (Island Study Linking Ageing and Neurodegenerative Disease)

**DOI:** 10.1002/alz.084164

**Published:** 2025-01-09

**Authors:** Eddy Roccati, Aidan Bindoff, Alex Kitsos, Hannah Fair, Jessica M Collins, Anna E King, Kathleen Doherty, Jane E Alty, James C Vickers

**Affiliations:** ^1^ Wicking Dementia Research and Education Centre, University of Tasmania, Hobart, TAS Australia; ^2^ Royal Hobart Hospital, Hobart, TAS Australia

## Abstract

**Background:**

People living in diverse rural areas have shown higher rates of Alzheimer’s disease and related dementias (ADRD) compared with their urban counterparts. Further, individuals in rural areas have higher rates of modifiable risk factors for ADRD, such as physical inactivity and alcohol misuse, that account for up to 40% of dementia cases. This study aimed to investigate the efficacy of a novel public health initiative to reduce dementia incidence in both urban and rural settings in Australia’s island state: Tasmania. We hypothesized that: 1) rural participants would display greater ADRD via risk factors, four‐year risk factor trajectories and phosphorylated tau (p‐tau) 181; and 2) both rural and urban participants would reduce their risk profiles over time.

**Method:**

Participants were from ISLAND (Island Study Linking Ageing and Neurodegenerative Disease) and were recruited from across Tasmania (n = 2,331, 70.9% female, average age 64.3 years, 26.6% APOE e4+). All participants were invited to complete yearly online surveys on background health, demographics, modifiable dementia risk via a custom Dementia Risk Profile (DRP), complete a free 6‐week Massive Open Online Course on Preventing Dementia (PD‐MOOC) and provide a blood sample for APOE genotyping and measurement of plasma p‐tau 181 (pg/mL). Multilevel longitudinal regression models assessed change in number and type of risk factors, with effects moderated by DRP and PD‐MOOC exposure.

**Result:**

Over four years of follow up, both urban and rural participants significantly reduced their modifiable risk factor profiles as measured via the DRP (p < 0.001). This benefit was greatest for participants who completed the PD‐MOOC. There was no significant difference in plasma p‐tau 181 (pg/mL) between urban and rural participants. Urban participants (n = 1,752; 75.2%) were significantly more likely to have a university qualification and be socioeconomically advantaged than rural (n = 579; 24.8%) participants.

**Conclusion:**

Our ISLAND public health dementia risk reduction initiative had a positive impact on modifiable risk factor adherence in both urban and rural Tasmanian Australians. This large‐scale cohort study shows that an online targeted public health campaign to reduce incidence and prevalence of ADRDs has the capacity to benefit both rural and urban populations.